# Supplementary material for: Face identification proficiency test designed using item response theory
Source: Behav Res Methods. 2023 Jun 9;56(3):1244–59. doi: 10.3758/s13428-023-02092-7 (PMC10991046; doi:10.3758/s13428-023-02092-7)
Supplement: Supplementary file 1 — (PDF 133 KB) [file 13428_2023_2092_MOESM1_ESM.pdf]

1 Supplemental Materials

1.1 Face recognition and identification tests

Table 1: Face recognition tests. Task Type: "M" denotes memory task: "P" denotes a perceptual task. Tests analyzed using "IRT" appear in blue.

Task Type	Test	Stimuli	Procedure
M	Cambridge Face Memory Test ?	72 items, 6 target faces	recognition (3-AFC)
M	Cambridge Face Memory Test (long form) ?	102 items	recognition (3-AFC)
M	Vanderbilt Face Matching Test ?	95 items, 2 catch items	recognition (3-AFC)
M	Penn Face Memory Test ?	20 targets, 20 foils	old/new
P	Glasgow Face Matching Test ?	168 image pairs	same/different (binary response)
P	Glasgow Face Matching Test (Short) ?	40 image pairs	same/different (binary response)
P	Expertise in Face Comparison Test ?	84 image pairs (half same identity)	same/different (response scale)
P	Person Identification Challenge Test ?	40 image pairs (half same identity)	same/different (response scale)
P	Kent Face Matching Test ?	200 match, 20 non-match	same/different (binary response)
P	Cambridge Face Perception Test ?	8 upright, 8 inverted	arrange 6 images based on target similarity
P	Yearbook Test ?	35 items	face similar to target
P	Face Identity Card Sorting Test ?	40 images (2 identities)	group images by identities

1.2 IRT Modeling Generalizability

On average, students' face identification ability is assumed to fall at the midpoint of the face proficiency distribution. Therefore, it is unclear if IRT modeling based on a student population can generalize to other populations (e.g., high ability groups such as super-recognizers or low ability groups such as prosopagnosics). As noted, testing special populations is beyond the scope of this study. However, to simulate a scenario of generalization, we conducted IRT modeling simulations. Our goal was to examine if IRT model built from lower-ability (higher-ability) participants provides accurate ability estimates of higher-ability (lower-ability) participants. To do this, participant ability was estimated by projecting data from participants of one ability group (e.g., high ability) onto an IRT model trained on a different ability group (e.g., low ability).

First, an IRT model was built using data from all 197 participants (Model "All"). Estimated participants' abilities served as the "ground-truth" for ability. Second, we divided participants into a higher-ability group ($N = 99$) and lower-ability group ($N = 98$), based on their percentage of correct scores (median split). Third, we built an IRT model with the lower ability group (Model "Low") and used the model to estimate the abilities of the higher-ability participants based on their raw responses. The projected abilities were compared to the ground truth abilities achieved from a model that included all participants. This step simulates a real-world situation where researchers would test face-identification examiners using an IRT model that is built from student populations. Fourth, we built an IRT model with the higher-ability group (Model "High") and estimated and analyzed the abilities of the lower-ability participants. This step simulates a real-world situation in which low-ability participants are tested using an IRT model derived from a student population.

Generalizability results appear in Figure 1 and show that IRT results created from all participants (Model "All") fall in between the model created from higher-ability individuals (Model "High") and the model created from lower-ability individuals (Model "Low"). The results can be interpreted from a correlational perspective and from the perspective of ability. From a correlational perspective, participant ability estimated from models built from all participants correlated positively with that achieved from models built from higher- and lower-ability participants[1]. From the perspective of ability, estimates of ability changed, based on the samples included. When the model included samples biased towards lower-ability individuals (top curve), the estimated abilities of the higher-end participants were higher than ground truth (middle curve). In contrast, when the model included samples biased towards the higher-ability individuals (bottom curve), the estimated abilities of the lower-end participants were lower than ground truth (middle curve).

These results justify support the validity of generalizing IRT modeling across different participant ability levels. IRT models build from specific ability groups

[1]Correlational values across all three models is .9997, p = 2.2e-16. Notably, this value is expected as results of a direct function of IRT properties and not a novel finding based on our data.

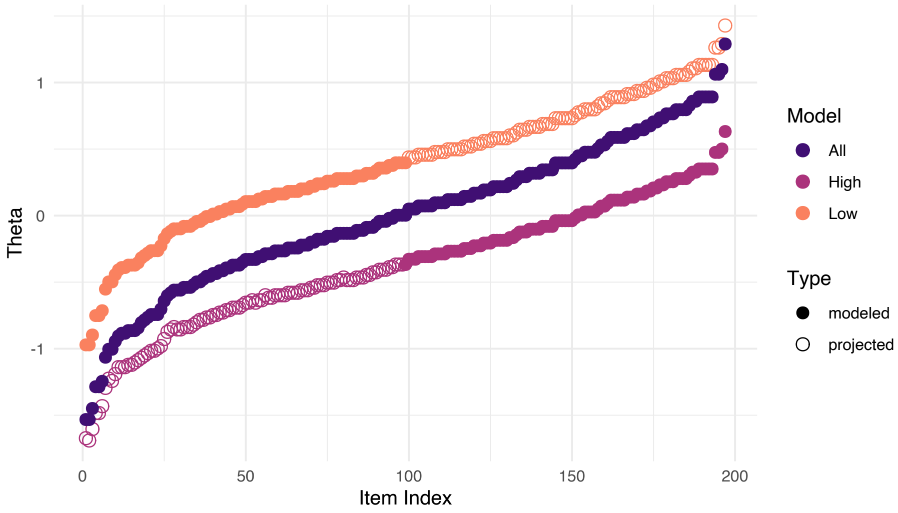

Figure 1: **IRT modeling generalization.** The middle dotted curve (purple) denotes the "ground-truth" abilities obtained from IRT models based on all participants. The bottom dotted curve (magenta) is derived from an IRT model built from the higher-half ability participants (determined by percent correct scores/ground-truth abilities). The top dotted curve (orange) is derived from an IRT model built from the lower-half ability participants (determined by percent correct scores/ground-truth abilities). Filled dots denotes ability estimated when the participant is included in the model. Hollow dots denotes ability estimated through projections (i.e., when the participant is excluded in the model).

can accurately measure another group regardless of differences in abilities. The IRT parameters provided in the current work can serve as benchmarks for evaluating future participants of wide-ranging ability.

References
